# Higher incidence of arrhythmia in COVID-19 than in other community-acquired pneumonia: possible role of concurrent therapies

**DOI:** 10.1186/s13054-021-03655-w

**Published:** 2021-07-22

**Authors:** Patrick M. Honore, Sebastien Redant, Thierry Preseau, Sofie Moorthamers, Keitiane Kaefer, Leonel Barreto Gutierrez, Rachid Attou, Andrea Gallerani, David De Bels

**Affiliations:** 1 Faculty of Medicine, ULB University, Brussels, Belgium; 2 ICU Department, Centre Hospitalier Universitaire Brugmann, Brugmann University Hospital, Place Van Gehuchtenplein, 4, 1020 Brussels, Belgium; 3 Emergency Department, Centre Hospitalier Universitaire Brugmann, Brussels, Belgium

In their recent meta-analysis, Liao et al. concluded that the incidence of arrhythmia was higher in COVID-19 than in other community-acquired pneumonia (CAP) (16.8% vs. 4.7%, 95% CI 2.4–8.9) [1, 2], with 2 out of 10 COVID-19 patients dying after developing arrhythmia [3]. Higher incidence rates of conduction disorders and premature contractions were found in COVID-19 patients, compared to other types of arrhythmias [1]. The authors noted that possible mechanisms of arrhythmia may include cardiac damage from metabolic disarray, hypoxia, neurohormonal or inflammatory stress and infection-related myocarditis in the setting of COVID-19 [4]. However, in the vast majority of the studies included, a substantial number of patients were receiving hydroxychloroquine [1], and sometimes azithromycin, and lopinavir/ritonavir [3]. Currently, there is no robust clinical evidence for a benefit associated with these drugs in the treatment of COVID-19, though most, if not all, are associated with the potential to prolong the QT interval, and induce ‘Torsades de Pointes,’ with a consequent risk of drug-induced sudden cardiac death [3]. We felt it important to point out that treatment with hydroxychloroquine in particular may have contributed to these arrhythmias in COVID-19 patients [1]. Given an estimated prevalence of 1 per 2000 of congenital long QT syndrome (LQTS) in the general population [5] and given the fact that it is generally considered to be significantly underdiagnosed, administration of QT interval prolonging drugs in COVID-19 patients may go some way to explain the increased incidence of arrhythmia. [5].

## Data Availability

Not applicable.

## Abbreviations

CAP: Community-acquired pneumonia
LQTS: Long QT syndrome

## References

[1] Liao SC, Shao SC, Cheng CW (2020). Incidence rate and clinical impacts of arrhythmia following COVID-19: a systematic review and meta-analysis of 17,435 patients. Crit Care.

[2] Corrales-Medina VF, Suh KN, Rose G (2011). Cardiac complications in patients with community-acquired pneumonia: a systematic review and meta-analysis of observational studies. PLoS Med.

[3] Carron J, Sharif Z, Hussein H (2021). Clinical guidance for navigating the QTc-prolonging and arrhythmogenic potential of pharmacotherapy during the COVID-19 pandemic. Ir J Med Sci.

[4] Driggin E, Madhavan MV, Bikdeli B (2020). Cardiovascular considerations for patients, health care workers, and health systems during the COVID-19 pandemic. J Am Coll Cardiol.

[5] Schwartz PJ, Stramba-Badiale M, Crotti L (2009). Prevalence of the congenital long QT syndrome. Circulation.

[6] Tisdale JE, Chung MK, Campbell KB, Hammadah M, Joglar JA, Leclerc J, Rajagopalan B, American Heart Association Clinical Pharmacology Committee of the Council on Clinical Cardiology and Council on Cardiovascular and Stroke Nursing (2020). Drug-induced arrhythmias: a scientific statement from the American heart association. Circulation.

[7] Bhimraj A, Morgan RL, Shumaker AH, Lavergne V, Baden L, Cheng VC, Edwards KM, Gandhi R, Gallagher J, Muller WJ, O'Horo JC, Shoham S, Murad MH, Mustafa RA, Sultan S, Falck-Ytter Y. Infectious diseases society of America guidelines on the treatment and management of patients with COVID-19. Infectious Diseases Society of America 2021; Version 4.3.0. https://www.idsociety.org/practice-guideline/covid-19-guideline-treatment-andmanagement/. Accessed 23 June 2021. 10.1093/cid/ciaa478 PMC7197612 32338708

[8] Pellicori P, Doolub G, Wong CM, Lee KS, Mangion K, Ahmad M, Berry C, Squire I, Lambiase PD, Lyon A, McConnachie A, Taylor RS, Cleland JG (2021). COVID-19 and its cardiovascular effects: a systematic review of prevalence studies. Cochrane Database Syst Rev.

