# Supplementary material for: Higher incidence of arrhythmia in COVID-19 than in other community-acquired pneumonia: possible role of concurrent therapies
Source: Crit Care. 2021 Jul 22;25:259. doi: 10.1186/s13054-021-03655-w (PMC8297605; doi:10.1186/s13054-021-03655-w)
Supplement: Related texts

# Reply: Authors’ response

**Authors:** Shu-Chen Liao, Shih-Chieh Shao, Ming-Jui Hung

**Affiliations:** 4 grid.454209.e 0000 0004 0639 2551 Department of Emergency Medicine, Keelung Chang Gung Memorial Hospital, Keelung, Taiwan; 5 grid.145695.a College of Medicine, Chang Gung University, Taoyuan, Taiwan; 6 grid.64523.36 0000 0004 0532 3255 School of Pharmacy, Institute of Clinical Pharmacy and Pharmaceutical Sciences, College of Medicine, National Cheng Kung University, Tainan, Taiwan; 7 grid.454209.e 0000 0004 0639 2551 Department of Pharmacy, Keelung Chang Gung Memorial Hospital, Keelung, Taiwan; 8 grid.454209.e 0000 0004 0639 2551 Section of Cardiology, Department of Internal Medicine, Keelung Chang Gung Memorial Hospital, No. 222, Maijin Rd., Anle Dist., Keelung, Taiwan; 9 grid.454209.e 0000 0004 0639 2551 Community Medicine Research Center, Keelung Chang Gung Memorial Hospital, Keelung, Taiwan

The authors appreciate Dr. Honore and his team’s valuable comments on our previous meta-analysis of arrhythmia in COVID-19 patients [1]. We agree with the viewpoint that higher incidence of arrhythmia in COVID-19 patients reported in prior literature could probably be explained by the complicated treatment [6]. However, the management strategies for COVID-19 are evolving quite rapidly, and some treatments with potential cardiac side effects, such as hydroxychloroquine, azithromycin and lopinavir/ritonavir, are not recommended under the current guidelines [7]. While existing systematic reviews and meta-analyses should be continually updated, our presented work maintains its emphasis on the clinical importance of monitoring arrhythmia to optimize patient outcomes during this pandemic [1, 8].
